# Kinetics and Mechanism of Epinephrine Autoxidation in the Presence of Plant Superoxide Inhibitors: A New Look at the Methodology of Using a Model System in Biological and Chemical Research

**DOI:** 10.3390/antiox12081530

**Published:** 2023-07-30

**Authors:** Vladimir Volkov, Anton Lobanov, Mikhail Voronkov, Timur Baygildiev, Vyacheslav Misin, Olga Tsivileva

**Affiliations:** 1 Laboratory of Chemistry of Antioxidants, N.M. Emanuel Institute of Biochemical Physics, Russian Academy of Sciences, 4 Kosygin Street, Moscow 119334, Russia; voronkov330363@yandex.ru (M.V.); misin@sky.chph.ras.ru (V.M.); 2 Department of Dynamics of Chemical and Biological Processes, N.N. Semenov Federal Research Center for Chemical Physics, Russian Academy of Sciences, 4 Kosygin Street, Moscow 119991, Russia; avlobanov@mail.ru; 3 Department of Chemistry, Lomonosov Moscow State University, 1-3 Leninskie Gory, Moscow 119991, Russia; t.baygildiev@vu.nl; 4 Amsterdam Institute for Life and Environment (A-LIFE), Vrije Universiteit Amsterdam, De Boelelaan 1085, 1081 HV Amsterdam, The Netherlands; 5 Institute of Biochemistry and Physiology of Plants and Microorganisms, Saratov Scientific Centre of the Russian Academy of Sciences (IBPPM RAS), 13 Prospekt Entuziastov, Saratov 410049, Russia

**Keywords:** autoxidation, epinephrine, chlorogenic acid, antioxidants, medicinal plants, superoxide

## Abstract

Superoxide is the primary active oxygen form produced in living organisms. Because of superoxide anion radical formation during epinephrine oxidation in alkaline medium, this system is offered in some works for antioxidant activity analysis, however, without enough physicochemical justification. Therefore, the task of developing reliable methods for analyzing the superoxide inhibition activity of various objects is very urgent. In this work, a kinetic model of epinephrine autoxidation in an alkaline medium in the presence of antioxidants of plant origin is proposed. The participation of chain reactions with long oxidation chains in this process is revealed. The limiting stage of the process is a one-electron reduction of oxygen by the anionic forms of the phenolic hydroxyls of epinephrine. The appearance of the absorption maximum at a wavelength of 347 nm during epinephrine autoxidation is associated with adrenolutin formation, which is confirmed by HPLC/UV/MS. No adduct formation between phenolic antioxidants and epinephrine oxidation products was found. The complex U-shaped character of epinephrine autoxidation rate dependence on the content of antioxidants in the reaction system was shown. The study of the kinetics of epinephrine autoxidation in the presence of an individual phenolic plant superoxide inhibitor, chlorogenic acid, was carried out for the first time. The inhibitory effect of yarrow, chamomile, and bur beggar-ticks plant extracts in the adrenaline system was examined.

## 1. Introduction

Reactions proceeding through radical mechanisms play an important role in the normal and pathological metabolism of all cellular life forms. Oxidative stress is known as the most important factor of the development of various chronic diseases [1], since many pathological processes are accompanied by the super reduction in the mitochondria electron transport chain, as well as the activation of enzymes that reduce oxygen by the one-electron scheme. One-electron oxygen reduction with the formation of superoxide is the main primary link in the initiation of free radical oxidation processes in living organisms [2]. Analysis of the antioxidant status changes in biological objects in response to various internal processes and external influences, and studying the activity of individual chemical compounds in reaction with reactive oxygen species gives important information in many physiological and biochemical investigations.

The metabolism of catecholamines includes superoxide formation. One of the systems for antioxidant activity (AOA) determinations is based on the inhibition of adrenaline autoxidation in an alkaline medium [3,4,5,6,7]. In addition to superoxide, one of the intermediate products in this system is semiquinone radicals, for which the ability to form covalent bonds with amino acids, peptides, and proteins has been revealed [8]. Adrenaline, like other catecholamines, in the presence of a number of enzymes or metal ions, such as Cu^2+^, Mn^2+^, Fe^2+^, Co^2+^, and Ni^2+^ [9,10], is capable of being oxidized under physiological conditions. These processes are important in the metabolic transformations of catecholamines. The sequence of chemical transformations is largely common to the different structures of catecholamines. The oxidation of catecholamines plays an important biological role, including melanin synthesis and the development of various pathological processes. The UV spectra of adrenaline oxidation products after incubation in plasma at 37 °C are identical to the spectra of adrenaline oxidation products in a strong basic buffer with maximum absorption at 347 nm [11]. 

**Figure 1:**
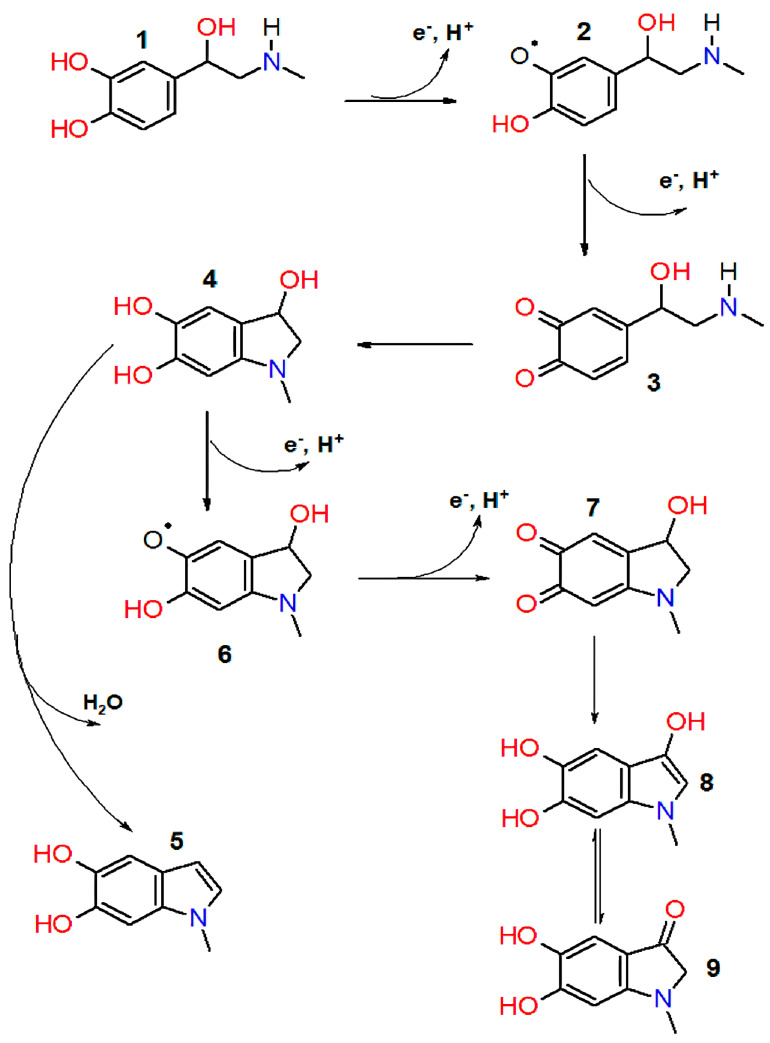
Scheme of epinephrine autoxidation process. 1—epinephrine; 2—epinephrine semiquinone; 3—adrenaline quinone; 4—leukoadrehochrome; 5—5,6—dihydroxy-N-methylindole; 6—adrenochrome semiquinone; 7—adrenochrome; 8, 9—adrenolutin (enol and ketone form).

Quinoid oxidation of adrenaline proceeds by a radical mechanism [12,13] through the one-electron reduction of oxygen and the formation of epinephrine semiquinone and epinephrine quinone, followed by a cyclization via the nitrogen atom nucleophilic addition to the aromatic ring, accompanied by the o-quinone group reduction. After that, leukoadrenochrome can be dehydrated to form 5,6-dihydroxy-N-methylindole or undergo further oxidation to form adrenochrome semiquinone and adrenochrome, which can be isomerized to adrenolutin (Figure 1). 

According to a review [14], 1–4 molecules of oxygen can be consumed per molecule of adrenaline, depending on the conditions of its oxidation. In a highly alkaline environment, up to three oxygen molecules are consumed per one adrenaline molecule. Thus, these data, in conjunction with the presence of two phenolic hydroxyls in the ortho position of adrenolutin, indicate that adrenolutin is capable of further oxidation. Change in the medium from highly alkaline to weakly acidic by adding HCl after oxidation does not lead to a shift in the wavelength of the absorption maximum λ = 347 nm of adrenaline oxidation products, which makes this band convenient in kinetic experiments [15]. Studies [16,17] have shown a significant effect of the reaction medium pH not only on the adrenaline autoxidation rate, but also on the color of the reaction products, which is apparently associated with different pathways of further conversions of adrenolutin [18]. In an acidified environment, the autoxidation of adrenaline proceeds slowly and is not accompanied by a solution color change for a long time without the addition of a catalyst. In an alkaline environment (pH value between 10 and 11), the solution acquires a lemon hue, and the oxidation process proceeds very quickly. At a pH of 7 to 8, the color of the solution turns pink, orange, red, and dark brown after 1, 3, 5, and 20 h, respectively.

The removal of the superoxide anion radical from the reaction system by adding superoxide dismutase slows down the accumulation of light-absorbing reaction products. This phenomenon is used for determining the activity of superoxide dismutase [3]. However, despite the fact that in some publications, this system is used to analyze the antioxidant activity of objects with a complex matrix of low-molecular-weight compounds [7,8], the technique has never been tested on individual low-molecular-weight antioxidants, with the exception of cysteine and ascorbic acid [19]. It should be noted that cysteine, like other thiols, is able to nucleophilic addition reactions in the presence of quinoid compounds [20]. Ascorbic acid is able to reduce adrenochrome [8]. This complicates the interpretation of the results obtained in the presence of these compounds since their reactivity in the system under study is not limited to antiradical activity.

In addition, the relationship between the inhibitory activity of low-molecular antioxidants and their concentration has not been investigated yet. The calculation algorithms were proceeded from an a priori assumption that this dependence is linear; meanwhile, antioxidants in high concentrations can change their activity up to the appearance of a prooxidant effect [21].

The measurements carried out by means of the high-performance liquid chromatography (HPLC) method show that the adrenaline content in blood plasma varies from 0.01 to 0.580 nmol/L [22]. S.L. Jewleet with co-authors presented data on the role of reactive oxygen species generated during the catecholamines catabolism, in particular, epinephrine autoxidation, in ischemia–reperfusion syndrome [23]. A number of researchers associate the development of schizophrenia with a disorder in the normal quinoid oxidation of adrenaline. According to the theory of J. Smythies and H. Osmond, adrenaline oxidation in this disease is accompanied by abnormally high adrenochrome formation caused by an excess of superoxide anion radicals, which, in turn, may be due to low superoxide dismutase activity in the body [24,25].

This research proposes a kinetic model of epinephrine autoxidation in an alkaline medium in the presence of antioxidants of plant origin.

## 2. Materials and Methods

### 2.1. Chlorogenic Acid Implementation

**Figure 2:**
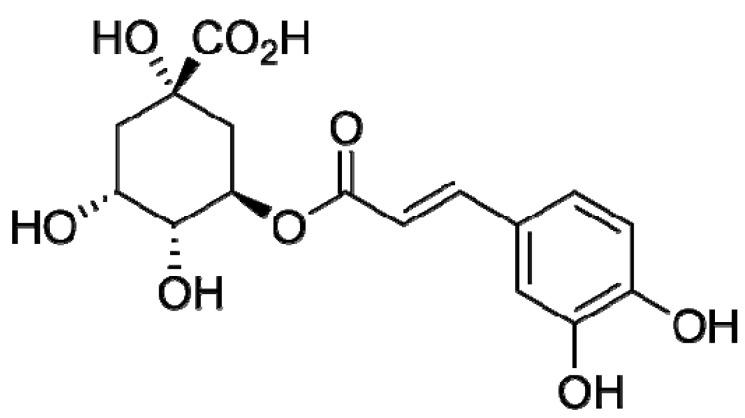
Chlorogenic acid structural formula.

Chlorogenic acid (Figure 2) was chosen as an individual model compound. Its activity toward the superoxide anion radical was the highest [26] in the series chlorogenic acid > caffeic acid > rutin > uric acid > genistin > trolox > glutathinone > acetyl cysteine. 

Chlorogenic acid belongs to a group of hydroxycinnamic acids; the products of its interaction with free radicals form C-C dimers [27], which also have antiradical properties. Chlorogenic acid (Fluka, Switzerland, ω = 97%) was dissolved in 96% ethanol to obtain a 1.58 mM solution.

### 2.2. Medicinal Plants Extracts Preparation

Plant raw material used was acquired from specialist suppliers: flowers of chamomile (*Matricaria chamomilla* L.), herb of bur beggar-ticks (*Bidens tripartita* L.), and herb of yarrow (*Achillea millefolium* L.). We chose water–propylene glycol extracts of medicinal plants as objects of natural origin with a complex matrix of biologically active compounds, which are widely used in cosmetic industry. Extraction of dry plant raw material of *M. chamomilla*, *B. tripartita* and *A. millefolium* was carried out with a 50% aqueous solution of 1,2-propylene glycol with a mass ratio of raw materials and extractant 1:19 in an Erlenmeyer flask at a temperature of 50 °C for 4 h with periodic stirring (raw material particle size 0.5–5 mm) [28]. The extract was filtered through a three-layer gauze filter and centrifuged at 5000 rpm for 20 min. 

### 2.3. Spectrophotometric Technique

A carbonate–hydrocarbonate buffer solution with pH = 10.65 was prepared as described in [29]. Sodium carbonate was preliminarily calcined for 1.5–2 h at a temperature of 200 °C. We used a 0.1% solution of epinephrine hydrochloride in ampoules produced by the Moscow Endocrine Plant (Russian Federation). A control experiment was carried out just before measurements. Experimental data were recorded using an Agilent Cary 60 spectrophotometer for 10 min in a 10 mm cuvette at 25 °C. Cuvette with a carbonate–hydrocarbonate buffer was placed into the comparison channel of the spectrophotometer. To prepare a series of reaction systems containing different volumes of chamomile, bur beggar-ticks, and yarrow extract, we mixed 4 mL of a carbonate–hydrocarbonate buffer, 0.2 mL of a 0.1% adrenaline solution, and aliquots of the investigated extracts in a series of increasing volumes. The total volume was adjusted with water to 4.26 mL. In experiments with chlorogenic acid, it was introduced into the reaction system in a volume of 0.001; 0.002; 0.01; 0.02; 0.05 milliliters (which corresponds to the final concentrations in the reaction system of 0.75; 1.5; 7.5; 15; 37.5 μM/L). In order to exclude influence of changes in the chemical structure of the analyzed objects on the dynamics of the reaction system’s absorbance, we also made experiments in which adrenaline was replaced with water.

### 2.4. Antiradical Antioxidant Analysis

Quantitative antiradical antioxidant analysis was carried out by implementing a spectrophotometric technique for examining the antioxidant (AO) interaction with 2,2-diphenyl-1-picrylhydrazyl (DPPH), a stable chromogenic free radical widely accepted as a tool for estimating radical-scavenging activity of AO [30,31]. Specifically, 2.7 mL of an ethanolic DPPH solution at a concentration of 8.1 × 10^−5^ M was added to the analyzed sample, and the reaction mixture volume was adjusted to 3.6 mL with ethanol and left to stay in a thermostat at *T* = 293 K for 30 min. Exactly after this time interval, the absorbance of tested solution was measured at a wavelength of 517 nm in a cuvette of 1 cm length light path. The sample volume was taken on the basis of the degree of DPPH radical conversion by the end of experiment desired by the targeted value of this degree within the range from 15 to 70%. The value of DPPH conversion degree was determined from the absorbance measurements followed by calculations using formula

*ζ* = 1 − *D*_exp_**/***D*_bl_, (1)

where *D*_exp_ is an absorbance of the tested solution detected in 30 min from the moment of the reaction start, *D*_bl_ is an absorbance of a blank solution, and ζ is a value of DPPH conversion degree detected in 30 min from the moment of mixing the reagents. To the blank sample, ethanol (96%, *v*/*v*) was added instead of the analyzed extract. The AO concentration in the tested extract was stated in terms of rutin equivalents (mol/L RE) using the formula

C_AO_ = [DPPH]_0_ *V*_syst_ *ζ*/(3.1 *V*_es_), (2)

where [DPPH]_0_ is a starting DPPH concentration in the reaction system (mol/L), *V*_syst_ is a reaction system volume (mL), *V*_es_ is an extract volume (mL) contained in the reaction system, and the quantity of 3.1 is the stoichiometric coefficient of DPPH inhibition by rutin under our experimental conditions. To state the AO concentration in terms of mg/L, these values were multiplied by a factor of 6.1 × 10^5^.

### 2.5. HPLC Analysis

High-performance liquid chromatography/tandem mass spectrometry (HPLC-MS/MS) data were obtained using a high-resolution LCMS-IT-TOF system (Shimadzu, Kyoto, Japan). Separation was performed on an Acclaim RSLC C_18_ column (150 mm × 2.1 mm, 2.2 μm, Thermo Fisher Scientific, Sunnyvale, CA, USA). The mobile phase consisted of a mixture of 0.1% (vol.) formic acid in water (A) and acetonitrile (B). Separation was carried out in the gradient elution mode. The flow rate of the mobile phase was 0.4 mL/min. The following gradient elution program was used: 0–4 min (1% B), 5–10.5 min (1% B–90% B), 10.5–13.5 min (90% B–90% B), 13.5–14.0 min (90% B–1% B), and 14.0–19.0 min (1% B–1% B). Column oven temperature was 35 °C. The volume of the injected sample was 10 μL. Operating conditions of the ion source of the mass spectrometer: positive and negative ESI modes (the mode of registration of positive and negative ions); interface voltage (voltage of the ionization source), 4500 V; nebulizing gas flow rate, 1.5 L∙min^−1^; CDL temperature, 275 °C; heat block temperature, 275 °C; drying gas pressure, 100 kPa; detector voltage, 1.55 V. Registration of mass chromatograms was carried out in the range of *m*/*z* 80–850, event time (time of one data collection cycle)—300 ms, repeat (number of repetitions)—3, and ion accumulation time (time of accumulation of ions in the ion trap)—30 ms. Mass calibration was performed prior to each analytical run, according to the manufacturer’s recommendations.

### 2.6. Quantitative Validation

The assessment of the acceptability of the measurement results was carried out in accordance with ISO 5725-6 standard.

## 3. Results and Discussion

### 3.1. Redox Status of the Systems with Chemicals and Plant Extracts

**Figure 3:**
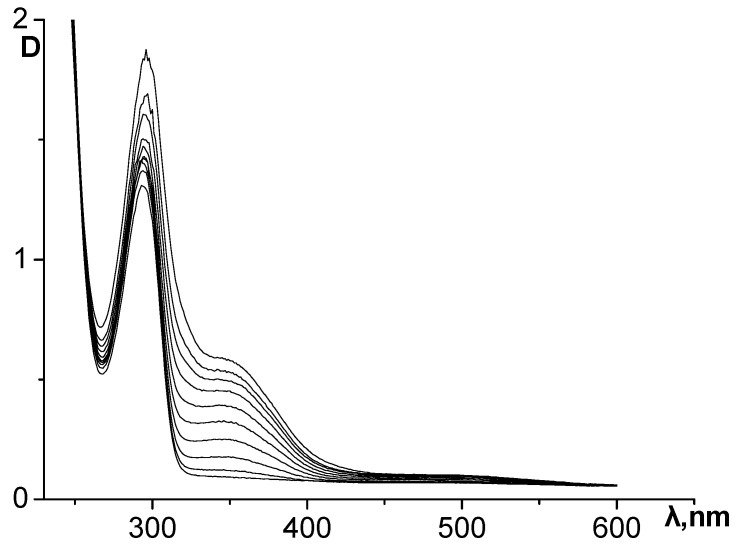
Dynamics of changes in the UV spectrum of the reaction system during epinephrine autoxidation in an alkaline carbonate–hydrocarbonate buffer. The curves were recorded at an interval of 30 s.

The dynamics of changes in the UV spectrum of the epinephrine oxidation reaction system without the addition of inhibitors are shown in Figure 3. During the formation of the reaction products, an intense increase in the absorbance of the absorption maxima at 293 and 347 nm is observed, as well as the appearance of insignificant absorption at about 480 nm.

**Figure 4:**
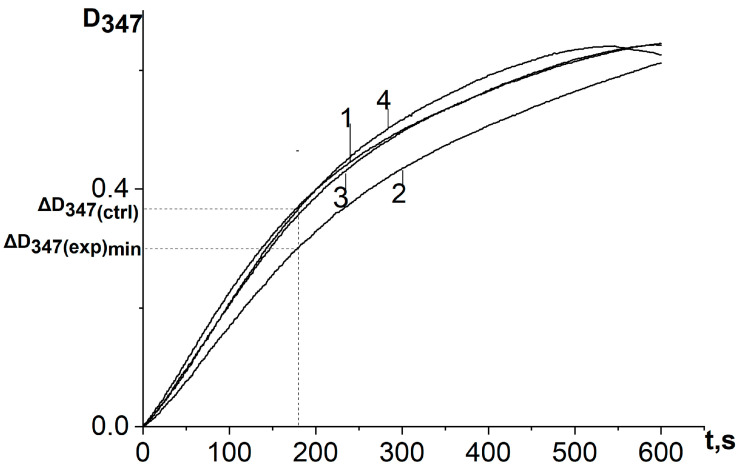
Kinetic curves of absorbance increase at a wavelength of 347 nm of the reaction system of adrenaline autoxidation in an alkaline medium (pH = 10.65) in the presence of chlorogenic acid in various concentrations: 1—control (no chlorogenic acid); 2—1.5 μmol/L; 3—5 μmol/L; 4—37.5 μmol/L. ΔD_347(exp)min_ is the absorbance increase in the reaction system for the first 3 min in the presence of antioxidant at a concentration corresponding to its maximum inhibitory efficiency; ΔD_347(ctrl)_ is the absorbance increase in the reaction system for the first 3 min in the absence of antioxidant. All ΔD_347_ values were calculated as the difference between the changes in absorbance over the first 3 min of the reaction in experiments carried out in the presence of adrenaline and in its absence (replacing adrenaline with an equal volume of distilled water).

**Figure 5:**
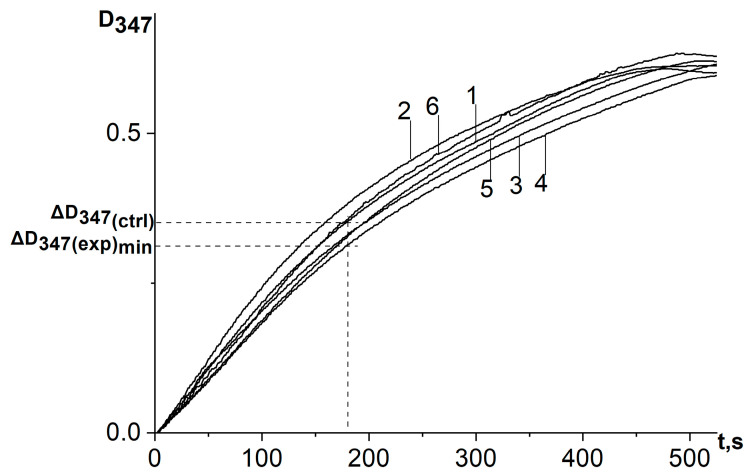
Kinetic curves of absorbance increase in adrenaline autoxidation reaction system in an alkaline medium (pH = 10.65) at a wavelength of 347 nm in the presence of a water–propylene glycol extract of chamomile (*Matricaria chamomilla* L.) added in different volumes: 1—control (no extract added); curves 2, 3, 4, 5, and 6 are referred to the volumes of 20 μL, 7 μL, 2 μL, 0.66 μL, and 0.23 μL, respectively. ΔD_347(exp)min_ is the absorbance increase in the reaction system for the first 3 min in the presence of antioxidant at a concentration corresponding to its maximum inhibitory efficiency; ΔD_347(ctrl)_ is the absorbance increase in the reaction system for the first 3 min in the absence of antioxidant. All ΔD_347_ values were calculated as the difference between the changes in absorbance over the first 3 min of the reaction in experiments carried out in the presence of adrenaline and in its absence (replacing adrenaline with an equal volume of distilled water).

When a water–propylene glycol extract of chamomile, bur beggar-ticks, or yarrow containing antioxidants of plant origin, as well as chlorogenic acid, was added to the reaction system, the process of adrenaline autoxidation slowed down. A decrease in the absorbance change rate at the analytical wavelength (347 nm) means inhibition of the adrenaline autoxidation products formation and the expression of antioxidant properties by the objects under study (Figure 4 and Figure 5). To determine the kinetic characteristics of adrenaline autoxidation inhibition, a three-minute interval was used from the moment of mixing the reagents. During this time, the difference between the absorbance values of the experiment and the control run reaches its maximum values, and the concentration of adrenaline is still far from being exhausted. It is important to note that, in contrary to the data [11], the curves do not show the induction period.

**Figure 6:**
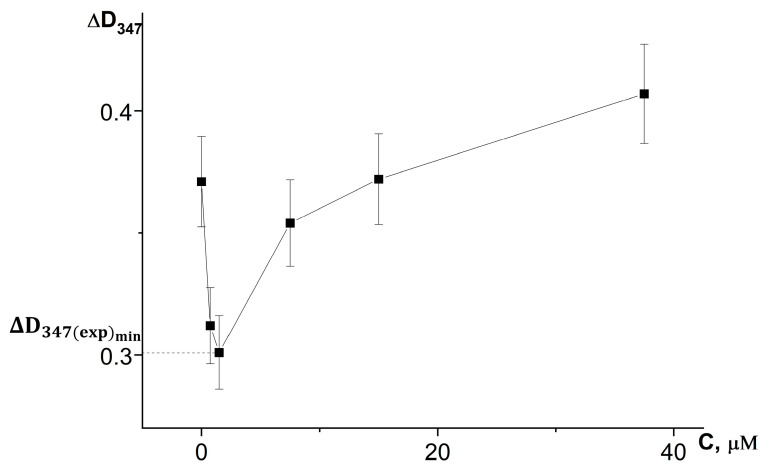
Dependence of the absorbance increase for the first 3 min at a wavelength of 347 nm (ΔD_347_) in the reaction of adrenaline autoxidation in an alkaline medium (pH = 10.65) on the concentration of chlorogenic acid. ΔD_347(exp)min_ is an increase in the absorbance for the first 3 min at a wavelength of 347 nm in the presence of chlorogenic acid in concentration corresponding to its maximum inhibitory efficiency. All the ΔD_347_ values were calculated as the difference between the changes in absorbance over the first 3 min of the reaction in experiments carried out in the presence of adrenaline and in its absence (replacing adrenaline with an equal volume of distilled water).

**Figure 7:**
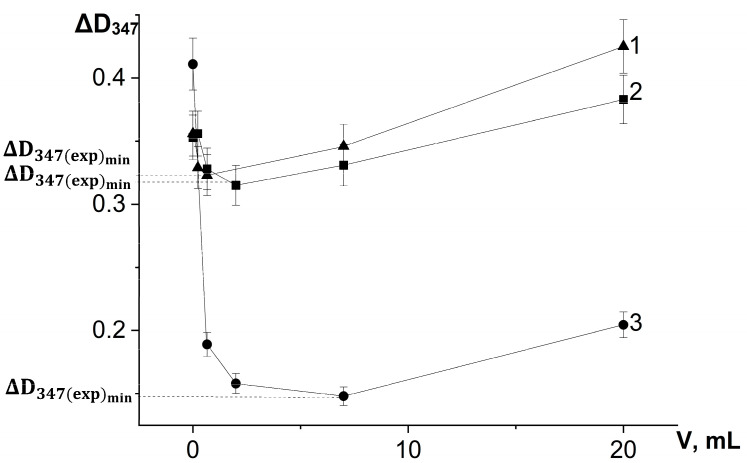
Dependence of the absorbance increase for the first 3 min at a wavelength of 347 nm (ΔD_347_) in the reaction of adrenaline autoxidation in an alkaline medium (pH = 10.65) on the volume of water-propylene glycol extracts added to the reaction mixture: 1—bur beggar-ticks (*Bidens tripartita* L.), 2—chamomile (*Matricaria chamomilla* L.), and 3—yarrow (*Achillea millefolium* L.). ΔD_347(exp)min_ is an increase in the absorbance for the first 3 min at a wavelength of 347 nm in the presence of chlorogenic acid in concentration corresponding to its maximum inhibitory efficiency. All the ΔD_347_ values were calculated as the difference between the changes in absorbance over the first 3 min of the reaction in experiments carried out in the presence of adrenaline and in its absence (replacing adrenaline with an equal volume of distilled water).

On the basis of a series of kinetic curves obtained by adding different amounts of test samples, we plotted the increase in absorbance during the first 3 min of the reaction on the concentration of chlorogenic acid in the reaction system (Figure 6) and the volumes of extracts introduced into the reaction system (Figure 7), taking into account changes in the absorbance of the samples themselves during the reaction.

Figure 6 and Figure 7 demonstrate that the expression of the antioxidant activity of substances in the adrenaline autoxidation system nonlinearly depends on their content in the reaction mixture. An increase in the content of antioxidants first leads to an increase in the inhibitory effect, and then, when the concentration corresponding to the maximum inhibitory activity is exceeded, a noticeable contribution of the prooxidant effect of the compounds begins, up to its dominance at high concentrations, which is consistent with theoretical concepts [21].

The data for yarrow extract and chlorogenic acid solution presented in Table 1 and Table 2 show that the absorbance of the samples themselves at the analytical wavelength in the absence of adrenaline in the buffer used does not undergo significant changes during the experiment.

For the investigated water–propylene glycol extracts, the maximum inhibitory effects were demonstrated by bur beggar-ticks and chamomile extracts when 2 µL was injected into the reaction system, and by yarrow extract when 7 µL was added (Figure 7). As can be seen in Figure 6, the maximum inhibitory efficiency of chlorogenic acid is observed at its concentration of 1.5 μmol/L in the reaction system. What is noteworthy is the fact that chlorogenic acid injected into the reaction system at a concentration more than two orders of magnitude lower than the concentration of adrenaline provides a significant decrease in the oxidation rate up to high conversion degrees of adrenaline. This demonstrates that the process of adrenaline autoxidation involves chain stages, and the length of these chains may be on the order of several tens of links. Since the reaction rate is highly dependent on pH, the rate-limiting stage of the process is the reaction of one-electron oxygen reduction by the anionic form of phenolic hydroxyl, the concentration of which increases proportionally to hydroxyl anion concentration in the system (Equations (3) and (4)):

HO-Ar-OH + OH^−^ ⇆ HO-Ar-O^−^ + H_2_O, (3)

HO-Ar-O^−^ + O_2_ ⇆ HO-Ar-O^●^ + O_2_^●−^ (4)

As a result, the superoxide formed in this way easily interacts with adrenaline to form adrenaline semiquinone, and then adrenaline semiquinone interacts with molecular oxygen to form a new superoxide radical (Equations (5) and (6)):

HO-Ar-OH + O_2_^●−^ → ^●^O-Ar-O^−^ + H_2_O_2_ (5)

^●^O-Ar-O^−^ + O_2_ → O=Ar=O + O_2_^●−^ (6)

Because chlorogenic acid and adrenaline have a similar structure of the phenolic parts of the molecules with two OH-groups in the ortho position, it is obvious that chlorogenic acid can also take part in the Reactions (3)–(6). To show the antioxidative properties in this system, polyphenol must have higher rate constant than adrenaline in the reaction with superoxide (Equation (5)) and lower rate constant with molecular oxygen (Equation (4)), and the semiquinone radical formed from polyphenol must have a low ability of radical chain propagation.

The rate constants of reactions (Equations (5) and (6)) can be evaluated by about 10^4^ and 10^5^ M^−1^ × s^−1^, respectively [8]. The observed rate constant *k* (Equation (7)) of the whole process under the conditions of our experiment can be calculated according to the law of mass action because we know the initial concentration of adrenaline C_0_ = 2.6 × 10^−4^ M, and the equilibrium concentration of oxygen C_O2_ in an aqueous solution at 25 °C is 2.51 × 10^−4^ M (without taking into account the salting-out effect). The initial reaction rate *ω*_0_ calculated from the initiation slope of the control experiment kinetic curve was 8.7 × 10^−7^ M.

*k* = *ω*_0_/(C_0_ × C_O_2__) = 13.2 M^−1^s^−1^ (7)

This value is significantly lower than the above-mentioned rate constants of reactions (Equations (5) and (6)). This confirms that the initiation of oxidation chains during the transfer of electrons from the anionic form of phenolic hydroxyl to molecular oxygen is the limiting stage that determines the observed rate of the whole process of adrenaline autoxidation. The above conclusions also explain the mechanism of catalysis of adrenaline autoxidation by variable valence metal ions since they are able to participate in one-electron oxygen reduction. Apparently, according to the same chain mechanism, the stage of oxidation of leukoadrenochrome to adrenochrome occurs.

**Figure 8:**
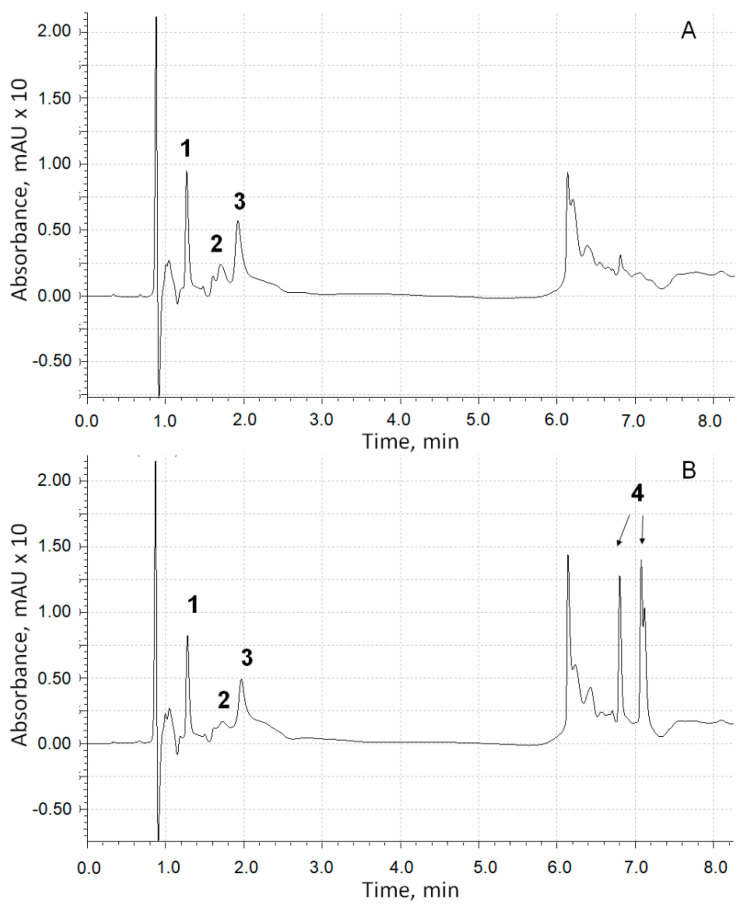
The UV chromatographic profiles at 254 nm of adrenaline autoxidation reaction system without chlorogenic acid addition (**A**) and in its presence (**B**): (1)—adrenaline; (2)—adrenochrome; (3)—adrenolutin; (4)—chlorogenic acid (two isomeric forms).

To reveal whether the inhibition of the formation of adrenaline oxidation products absorbing at a wavelength of 347 nm is a consequence of the formation of adducts with chlorogenic acid, we conducted a chromatographic analysis of the reaction products. UV spectrometric detection proceeded at a wavelength of 254 nm (on which all aromatic compounds absorb). Chromatographic profiles of the components of the epinephrine oxidation reaction system in the absence (Figure 8A) and in the presence (Figure 8B) of 4 × 10^−5^ M chlorogenic acid after a five-minute exposure and subsequent neutralization with hydrochloric acid to pH = 5.5 were registered.

Obviously, the profile in Figure 8B does not contain additional peaks, with the exception of those related to chlorogenic acid isomers, which forms negatively charged ions [M-H]^−^ in an MS assay with *m*/*z* = 353.09 (peaks 4). Thus, the version about the formation of adducts of adrenaline autoxidation products with chlorogenic acid is not confirmed.

**Figure 9:**
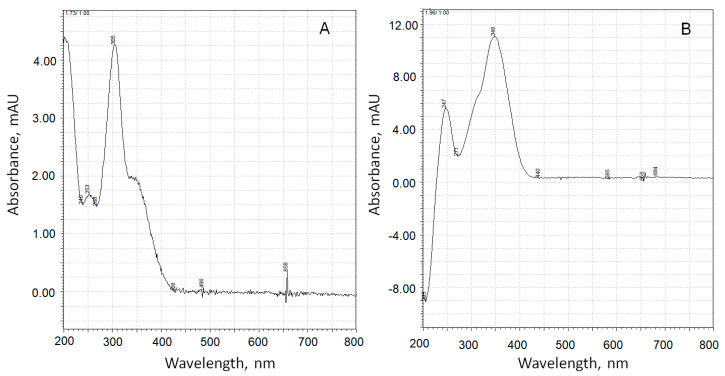
UV spectra of adrenaline autoxidation products with retention times of 1.73 min (**A**) and 1.96 min (**B**).

In the scientific literature, there were no experimentally confirmed data on the identification of the chemical structure of the adrenaline oxidation product, which is responsible for the increase in absorbance at 347 nm. To answer this question, data obtained by means of chromatography with mass detection and three-dimensional ultraviolet (UV) spectrometric detection were used. Figure 8 shows a peak corresponding to adrenaline (peak 1, *m*/*z* of adduct with proton is 184.10) and two products of its oxidation (peaks 2 and 3), the UV spectra of which are shown in Figure 9. 

The substance corresponding to chromatographic peak 3 forms a positively charged sodium adduct with *m*/*z* = 202.04 and may be referred to adrenolutin, which has a monoisotopic mass of 179.058. Other peaks in the chromatographic profile (Figure 8A) have UV spectra with no pronounced maxima at wavelengths above 250 nm, with the exception of a peak corresponding to a retention time of 1.05 min with a wide maximum at 261 nm in its UV spectrum. Peaks with retention times of 6.14 and 6.23 min are absent in the ionic current chromatographic profile.

According to quantum chemical calculations [16], adrenochrome has an absorption band at 293 nm, and adrenolutin has a calculated band at 369 nm. Since there are no other chromatographic peaks with UV maxima close to the above-mentioned data, it is obvious that peak 2 with the spectrum in Figure 9A should be attributed to adrenochrome and peak 3 with the spectrum in Figure 9B to adrenolutin. The absence in the spectrum of the peak 2 compound of the absorption band at about 480 nm is due to the acidic reaction of the chromatographic eluent. In our experiments, it was demonstrated that when the solution of adrenaline oxidation products was transferred from an alkaline medium to an acidic one, the 480 nm band disappeared.

In the works of our predecessors [4,5,6,7], relative AOA was assessed by the formula:

AOA = {1 − (ΔD_(exp)_/ΔD_(ctrl)_)} × 100%, (8)

where ΔD_exp_ and ΔD_ctrl_ are an increase in the absorbance of the reaction system at a wavelength of 347 nm for a selected time from the start of the reaction in the presence and absence of the analyzed samples.

In agreement with the complex non-monotonic concentration dependence of the inhibitory effect discovered by us, this formula must be modified as follows:

AOA = [ΔD_347(ctrl)_ − ΔD_347(exp)min_]/ΔD_347(ctrl)_ × 100%, (9)

where ΔD_347(exp)min_ is the absorbance increase in the reaction system for the first 3 min in the presence of antioxidant at a concentration corresponding to its maximum inhibitory efficiency, ΔD_347(ctrl)_ is the absorbance increase in the reaction system for the first 3 min in the absence of antioxidant. Table 3 shows the calculated antioxidant activity parameters for the studied samples.

A comparison of data on AOA and C_AO_ shows that the offered system of inhibited adrenaline autoxidation provides fundamentally new information compared to the data of quantitative analysis by the DPPH method. The inhibitory effect of yarrow extract in the adrenaline system increases up to AO concentrations equivalent to 2 mg/L or 3.2 µM of rutin, while at such concentrations of AO in chamomile and bur beggar-ticks extracts, the prooxidant effect prevails over the antioxidant one. It is interesting that the antioxidant concentration in yarrow extract corresponding to its maximum inhibitory effect is almost equivalent to the concentration of the maximum inhibitory effect of chlorogenic acid, which is 1.6 µM.

As can be found in the literature sources, the phenolic antioxidant compositions of all these three plant species are mostly similar [32,33,34]. There are chlorogenic acid and caffeic acid, rutin, apigenin, quercetin, and luteolin and their derivatives in aqueous and others extracts of these plants. Despite this similarity, there are differences in the dominant compounds: apigenin 7-glucoside and apigenin in *Matricaria chamomilla*; luteolin 7-O-glucoside in *Bidens tripartite*; and dicaffeoylquinic and pure luteolin in *Achillea milefolium*. Unfortunately, we can see that these data do not explain the high AOA value of yarrow water–propylene glycol extract in comparison with other extracts and pure chlorogenic acid.

### 3.2. Acidity Dependence of Polyphenols AOA

Epinephrine oxidation in acidic conditions proceeds very slowly. High pH values are to be used in this system to enhance both the oxidation rate of epinephrine and the superoxide formation rate. Phenolic antioxidants like chlorogenic acid and others existing in plant extracts react with free radicals via hydrogen atom abstraction (HAT) or sequential proton loss electron transfer mechanism (SPLET), but the latter is predominant in solvents that form hydrogen bonds at basic, neutral, or even weak acidic pHs [35]. That is why there are no sufficient changes in the mechanisms of antioxidant effects of phenolic compounds in the pH area from 10.65 to pH values of blood plasma and skin. Of course, at high pH values, due to the high ionization degree of polyphenols, their prooxidant effects may become noticeable at lower concentrations than at neutral pHs, but the aim of this study as for antioxidant/prooxidant activity concentration dependence was to choose the correct conditions for comparing the antioxidant activity of different objects.

### 3.3. Study of Extraction Efficiency of Water–Propylene Glycol Mixture

**Figure 10:**
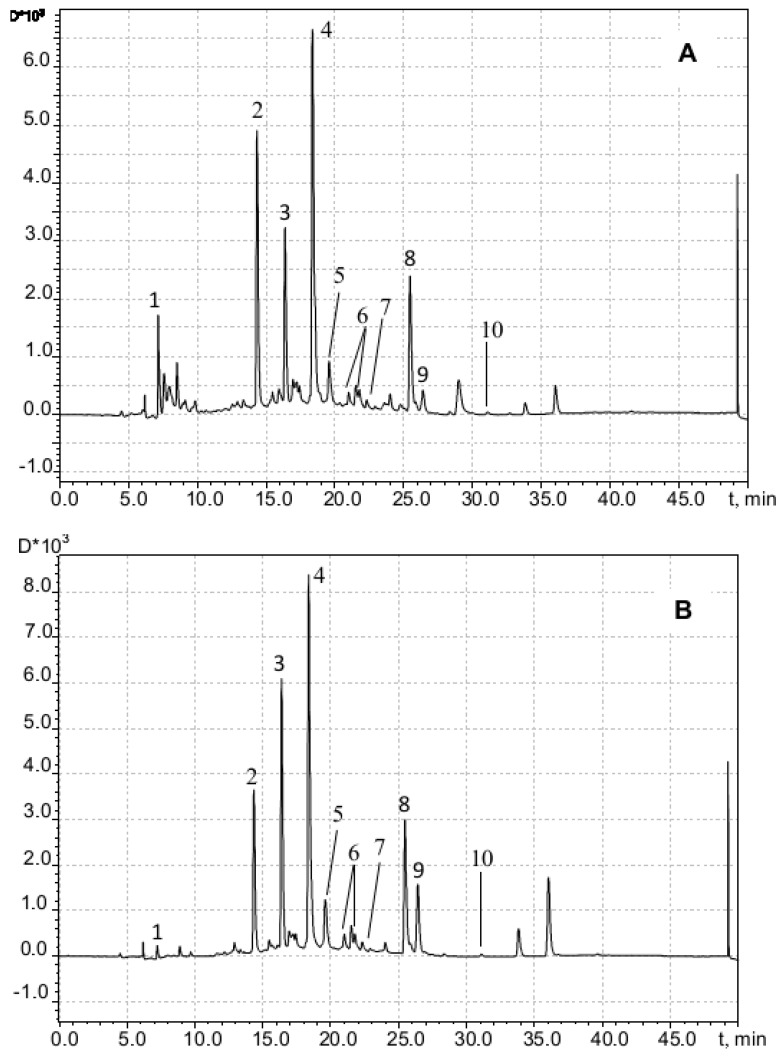
Chromatogram of chamomile flowers extract (water-propylene glycol, 50%) recorded at 254 nm (**A**) and 330 nm (**B**): (1) mononuclear phenolic diglycoside (presumably C_22_H_30_O_12_); (2,3) cis and trans isomers, respectively, of 2-glucopyranosyloxy-4-methoxycinnamic acid; (4) apigenin-7-*O*-glucoside; (5) apigenin-7-*O*-6-malonyl-glucoside; (6) apigenin-7-*O*-acetyl-glucoside; (7) acetyl-malonyl-apigenin-7-*O*-glucoside; (8) apigenin; (9) 7-methoxycoumarin; (10) 5,4′-dioxi-3,6,7,3′-tetramethoxyflavone.

Although water–propylene glycol and purely propylene glycolic phytoextracts have found numerous applications in the cosmetic industry, an overwhelming majority of published research lacks data on the optimal percentage composition and extracting power of water–propylene glycol mixtures compared to aqueous–ethanolic ones. High-performance chromatographic (HPLC/UV/MS) determinations with the aqueous–propylene glycolic extracts from the *M. chamomilla* specimen used in this work detect apigenin and its 7-O-glucopyranoside derivatives with the differently positioned acyl group (apigenin-7-O-glucopyranoside (cosmosiin), malonyl-apigenin-7-O-glucopyranoside, acetyl-apigenin-7-O-glucopyranoside, and acetyl-malonyl-apigenin-7-O-glucopyranoside) and 5,4′-dioxy-3,6,7,3′-tetramethoxyflavone. In these assays, the retention time values of 14.33 min and 16.36 min correspond to the compounds with the brutto formula C_16_H_20_O_9_ (mass of molecular ion [M − H]^−^ is 355.1016, Δ = −5.3 ppm). Taking into account the molecular ion [M + Na]^+^ mass equal to 379.1003 and the mass quantities for the fragmentation products (177.0542 and 195.0649), all that together could bring us to the conclusion that the aforesaid substances are monoglucosides of either ferulic acid or its isomers [36]. HPLC coupled with nuclear magnetic resonance spectroscopy (HPLC-NMR) method implementation results [37] demonstrate that the chamomile plant material’s water–ethanol extracts contain both cis and trans isomers of 2-hydroxy-4-methoxycinnamic acid 2-O-glucopyranoside. Therewith, 2-hydroxy-4-methoxycinnamic acid, an isomer of ferulic acid (which is 4-hydroxy-3-methoxycinnamate), could undergo intramolecular cyclization with the corresponding coumarin ring closure to yield 7-methoxycoumarin. A comparison of our findings obtained from the peaks 2, 3, and 9 UV and MS spectra (Figure 10) with the results gained by Weber et al. in 2008 [38] appears to be indicative of the presence of those compounds in our aqueous–propylene glycolic extract.

With allowance for the presumed formula C_22_H_30_O_12_ (molecular ion [M + H]^+^ mass equal to 487.1791, Δ = −3.9 ppm) and the absorption maximum in UV spectra at 280 nm, peak 1 (retention time 7.2 min) could be attributed to diglucoside of a mononuclear phenolic compound. As the major components of water–ethanolic extracts from the *Matricaria chamomilla* plant material, apigenin and its derivatives are referred to in the scientific literature [38], which appears to be true also for the aqueous–propylene glycolic extract from the chamomile plant that we studied in this work. Thus, both aqueous solvents and mixtures with propylene glycol or ethanol essentially yield the same major extractable components when serving as the extracting agents to treat this plant material. Moreover, based on these results, it can be assumed that the studied water–propylene glycol extracts are similar in composition to the water–ethanol extracts of the corresponding medicinal plants. 

## 4. Conclusions

The results of our study prove that the autoxidation of adrenaline in an alkaline medium is a multistage process with long oxidation chains driven by superoxide. The observed rate of the process is determined by the rate of one-electron reduction of oxygen by anionic forms of phenolic hydroxyls of adrenaline and the oxidation chain termination rate, including termination of antioxidants injected into the reaction system. Changes in the intensity of light absorption at a wavelength of 347 nm during adrenaline autoxidation in an alkaline medium are associated with the formation of adrenolutin. Thus, our research proves that the parameters determined by this method really are associated with the antioxidant activity of the analyzed objects.

An analysis of the antioxidant activity of any object in a system based on the autoxidation of adrenaline must be carried out in a wide range of concentrations due to the complex character of the concentration dependence of the inhibitory effects of antioxidants. An increase in the antioxidant concentration first leads to an increase in its inhibitory properties, and then, upon reaching a specific value for each object, the antioxidant effect is replaced by a prooxidant effect. As a result, the curve of absorbance increases in dependence on antioxidant concentration for a fixed period of time from the start of the reaction and has a “U-shaped” character. The descending branch of the curve corresponds to an increase in antioxidant activity; the minimum point corresponds to the concentration of antioxidant at which the maximum antioxidant activity is observed; and the ascending branch is an area of significant prooxidant effects. Underestimating this circumstance can lead to erroneous results in a comparative analysis of the antioxidant activity of several samples. For example, if the concentration of antioxidants in one of them corresponds to the descending branch of the U-shaped curve and in the other to the ascending branch, the antioxidant properties of the second would be significantly underestimated. Thus, the proposed modification of the method allows us not only to carry out a more correct comparison of the activity of samples with respect to superoxide but also to draw conclusions about the maximum effective concentration of a sample with antioxidant properties.

The fact that such important natural antioxidants as thiols interact with non-radical intermediate products of catecholamine oxidation complicates the interpretation of the research data obtained for biological samples by this system and necessitates additional studies. Nevertheless, according to our studies using chlorogenic acid as an example, phenolic antioxidants do not form adducts with adrenaline oxidation products. That is, inhibition occurs only through interaction with free radicals.

The method requires the researcher to have experience conducting kinetic experiments and to perform the manipulations accurately.

In the study [5], it was proposed to take the duration of the induction period of the autoxidation reaction as a parameter of antioxidant activity. However, our experiments on adrenaline autoxidation in the presence of chlorogenic acid, a substance with activity against superoxide anion radicals proved by EPR, demonstrated that both uninhibited and inhibited adrenaline autoxidation did not have an induction period.

Surprisingly, the maximum inhibitory efficacy of the antioxidant complex of the water–propylene glycol extract of yarrow turned out to be more than three times higher than that of pure chlorogenic acid.

## Figures and Tables

**Table 1:** Changes in absorbance of the reaction system, containing adrenaline (D_347_) and not containing adrenaline (ΔD_347(no adrenaline)_), at a wavelength of 347 nm (carbonate–hydrocarbonate buffer, pH = 10.65) in 3 min from the beginning of the reaction in the presence of water–propylene glycol extract of yarrow (*Achillea millefolium* L.). V_extract_ is volume of yarrow extract added to the reaction system.

V_extract_, µL	D_347_ (Buffer Solution, Adrenaline, and Extract)	ΔD_347(no adrenaline)_ (Buffer Solution and Extract)	ΔD_347_ = D_347-_ ΔD_347(no adrenaline)_
20	0.230 ± 0.011	0.025 ± 0.005	0.204 ± 0.010
7	0.146 ± 0.007	−0.002	0.148 ± 0.007
2	0.172 ± 0.009	0.002	0.170 ± 0.009
0.66	0.188 ± 0.009	−0.001	0.189 ± 0.009

**Table 2:** Changes in absorbance of the reaction system, containing adrenaline (D_347_) and not containing adrenaline (ΔD_347(no adrenaline)_), at a wavelength of 347 nm (carbonate–hydrocarbonate buffer, pH = 10.65) in 3 min from the beginning of reaction in the presence of chlorogenic acid. C is concentration of cholorogenic acid in the reaction system.

C_,_ µmol	D_347_ (Buffer Solution, Adrenaline, and Chlorogenic Acid)	ΔD_347(no adrenaline)_ (Buffer Solution and Chlorogenic Acid)	ΔD_347_= D_347-_ ΔD_347(no adrenaline)_
0.75	0.311 ± 0.016	−0.001	0.312 ± 0.016
1.5	0.301 ± 0.015	−0.002	0.303 ± 0.015
7.5	0.351 ± 0.018	−0.003	0.354 ± 0.018
15	0.357 ± 0.018	−0.015 ± 0.005	0.372 ± 0.019
37.5	0.367 ± 0.019	−0.04 ± 0.005	0.407 ± 0.020

**Table 3:** Values of antioxidant activity of the studied objects.

Object	ΔD_347(ctrl)_	ΔD_347(exp)min_	AOA, %	C_AO_ in Extract by DPPH, mg/L RE	C_AO_ Corresponding to Maximum Inhibitory Effect, µM RE
Water–propylene glycol extract of yarrow (*Achillea millefolium* L.)	0.411 ± 0.020	0.148 ±0.007	64.0 ± 3.2	1240 ± 60	3.2
Water–propylene glycol extract of camomile (*Matricaria chamomilla* L.)	0.381 ± 0.019	0.318 ± 0.016	16.5 ± 0.8	148 ± 11	0.07
Water–propylene glycol extract of bur beggar-ticks (*Bidens tripartita* L).	0.356 ± 0.018	0.323 ± 0.016	9.3 ± 0.5	262 ± 19	0.12
Chlorogenic acid solution	0.371 ± 0.018	0.301 ± 0.015	18.0 ± 0.9		1.6

## Data Availability

Data supporting reported results can be found within the article.

## Abbreviations

AO	antioxidant
AOA	antioxidant activity
DPPH	2,2-diphenyl-1-picrylhydrazyl
HAT	hydrogen atom abstraction
HPLC	high-performance liquid chromatography
HPLC-MS/MS	high-performance liquid chromatography/tandem mass spectrometry
LC	liquid chromatography
MS	mass-spectrometry
NMR	nuclear magnetic resonance
RE	rutin equivalents
SPLET	sequential proton loss—electron transfer mechanism
UV	ultraviolet
